# Mitochondrial protein import stress in Parkinson’s disease: the TOM/TIM–PINK1/Parkin–DELE1/HRI axis linking mitochondrial proteostasis to neuronal death

**DOI:** 10.3389/fcell.2026.1935788

**Published:** 2026-09-28

**Authors:** Bo Pang, Xin Li, Yunlong Sun, Lin Ding

**Affiliations:** 1 College of Pharmacy, Jining Medical University, Rizhao, Shandong, China; 2 Institute of Pharmacy, Shandong University of Traditional Chinese Medicine, Shandong, China

**Keywords:** DELE1, HRI, integrated stress response, mitochondrial protein import, mitophagy, parkin, Parkinson’s disease, PINK1

## Abstract

Mitochondrial dysfunction is a hallmark of Parkinson’s disease (PD), but how impaired mitochondrial homeostasis drives the selective death of dopaminergic neurons is still poorly understood. At the center of this problem lies the mitochondrial protein import machinery—the TOM and TIM23 translocases—which doubles as a stress-sensing hub that coordinates two distinct quality control pathways. When import is compromised, the kinase PINK1 accumulates on the mitochondrial surface, recruits Parkin to ubiquitinate outer membrane proteins, and triggers mitophagy. Meanwhile, the stress sensor DELE1 is cleaved and released to the cytosol, where it activates the HRI kinase to phosphorylate eIF2α and engage the integrated stress response (ISR). These two pathways are not independent. Recent work shows they engage in antagonistic crosstalk: the DELE1–HRI–ISR axis suppresses PINK1/Parkin-dependent mitophagy by preserving mitochondrial import efficiency, acting as a molecular brake on organelle clearance. The balance between these pathways is governed by the SIFI E3 ubiquitin ligase complex, which targets both DELE1 and HRI for degradation. Disruption of SIFI causes progressive neurodegeneration in humans. Here we synthesize the structural, biochemical, and genetic evidence that defines the TOM/TIM–PINK1/Parkin–DELE1/HRI signaling axis, examine how its dysregulation contributes to dopaminergic neuron vulnerability, and evaluate therapeutic strategies targeting mitochondrial import stress in PD.

## 1 Introduction

Parkinson’s disease is the second most common neurodegenerative disorder, affecting roughly 1% of people over 60 (Bloem et al., 2021). Its pathological hallmarks are progressive loss of dopaminergic neurons in the substantia nigra pars compacta and accumulation of α-synuclein-rich Lewy bodies. Most PD cases are sporadic, but monogenic forms—mutations in *SNCA*, *LRRK2*, *PINK1*, *PRKN* (Parkin), *DJ-1*, and *ATP13A2*—have revealed key molecular pathways that drive neuronal vulnerability (Blauwendraat et al., 2020). Several of these genes converge on mitochondrial quality control.

Mitochondria are essential for neuronal survival: they supply ATP via oxidative phosphorylation, buffer cytosolic calcium, and regulate apoptosis. Nearly all of the ∼1,200 mitochondrial proteins are nuclear-encoded, synthesized on cytosolic ribosomes, and imported through dedicated translocases (Pfanner et al., 2019). The TOM (translocase of the outer membrane) complex is the universal entry gate; the TIM23 (translocase of the inner membrane) complex mediates translocation into and across the inner membrane. For long-lived, highly polarized cells like dopaminergic neurons—whose extensive axonal arbors and sustained bioenergetic demands place relentless pressure on mitochondrial proteostasis—the efficiency of this import process is especially critical (Nar et al., 2024).

The discovery that PINK1, a mitochondrial kinase linked to early-onset PD, acts as a sensor of import competence changed how we think about mitochondrial damage detection (Narendra et al., 2010; Jin et al., 2010). In healthy mitochondria, PINK1 is imported through TOM-TIM23, processed by mitochondrial proteases, and degraded. When import fails—whether from depolarization, misfolded proteins, or translocase dysfunction—PINK1 accumulates on the outer membrane, phosphorylates ubiquitin, and recruits Parkin to initiate mitophagy (Nar et al., 2024; Pickles et al., 2018). A second, parallel sensing mechanism was uncovered more recently: the mitochondrial protein DELE1 is also dependent on TOM-TIM23 import for its constitutive turnover. When import is disrupted, a cleaved C-terminal fragment of DELE1 escapes to the cytosol and activates the HRI (EIF2AK1) kinase, triggering the ISR (Fessler et al., 2020; Guo et al., 2020; Yang et al., 2023).

Both pathways originate from the same event—mitochondrial import failure—but drive opposite outcomes: organelle elimination versus transcriptional reprogramming. Work published between 2023 and 2025 established that these pathways intersect. The DELE1–HRI–ISR axis suppresses PINK1-dependent mitophagy by preserving mitochondrial import efficiency through translational control (Singh et al., 2025; Yang et al., 2026). The SIFI E3 ubiquitin ligase complex was identified as the master silencer of this axis: it targets both DELE1 and HRI for degradation, and mutations in its UBR4 subunit cause progressive ataxia and dementia (Haakonsen et al., 2024).

This review provides a synthesis of the TOM/TIM–PINK1/Parkin–DELE1/HRI axis in PD. We describe the import machinery as the foundation for stress sensing, examine the PINK1/Parkin and DELE1/HRI branches, and analyze their crosstalk. We then discuss the SIFI silencing complex, the implications for selective dopaminergic neuron vulnerability, and emerging therapeutic strategies. The principal molecular components, pivotal mechanistic studies, and PD-associated mutations and therapeutic targets discussed throughout this review are summarized in Tables 1–3, respectively.

**TABLE 1:** Key components of the TOM/TIM–PINK1/Parkin–DELE1/HRI signaling axis.

Component	Localization	Molecular function	PD relevance
TOMM40	OMM	Core β-barrel pore of TOM complex; universal entry gate	Required for PINK1 accumulation on damaged mitochondria
TOMM20	OMM	MTS receptor; directly binds PINK1 NT-CTE module; gates PINK1 activity	PD-associated PINK1 mutations disrupt TOMM20 binding; supercomplex impaired
TOMM7	OMM	Regulatory TOM subunit; stabilizes PINK1 on OMM	Essential for PINK1 retention and downstream mitophagy
TOMM5	OMM	Maintains PINK1–TOMM40 contacts during import arrest	Loss abrogates PINK1 accumulation without affecting general import
TIMM23	IMM	Core channel of TIM23 translocase	Required for PINK1 import; TIM23 ablation arrests PINK1 on TOM
TIMM17	IMM	TIM23 channel subunit; interacts with PARL and ROMO1	Governs PINK1 fate: PARL cleavage vs. matrix import
PINK1	OMM/IMM/Matrix	Ser/Thr kinase; sensor of import competence; phosphorylates ubiquitin (Ser65)	Loss-of-function mutations cause autosomal recessive PD (PARK6)
Parkin	Cytosol/OMM	E3 ubiquitin ligase; ubiquitinates OMM proteins to initiate mitophagy	Loss-of-function mutations cause early-onset PD (PARK2)
DELE1	Matrix/Cytosol	Stress sensor; TPR-domain protein; activates HRI upon import failure	Processing impaired by PD-associated HtrA2 G399S
HRI (EIF2AK1)	Cytosol	eIF2α kinase; activated by DELE1-S oligomers; triggers ISR	Negative regulator of PINK1/Parkin mitophagy
OMA1	IMM	Metalloprotease; cleaves DELE1 at His142	Cell-type-specific requirement for DELE1 processing
HtrA2	IMS	Serine protease; generates DELE1-VS	G399S linked to PD/essential tremor; impairs DELE1 processing
PARL	IMM	Rhomboid protease; cleaves PINK1 TMD at A103–F104	Required for normal PINK1 turnover
ROMO1	IMM	Proposed alternative TIMM17-associated partner that favors full PINK1 translocation into the matrix rather than PARL-mediated cleavage	May influence PINK1 matrix-import fate at the PARL-versus-ROMO1 branch point; direct relevance to PD remains to be established
SIFI (UBR4–KCMF1–CaM)	Cytosol	E3 ligase; degrades cDELE1 and HRI; silences mitochondrial ISR	UBR4 mutations cause ataxia and early-onset dementia
ISRIB	— (Pharmacological)	eIF2B activator; reverses translational effects of p-eIF2α	Enhances PINK1/Parkin mitophagy; preclinical tool compound

**TABLE 2:** Key studies (2020–2026) defining the crosstalk between mitochondrial import stress, PINK1/Parkin mitophagy, and the DELE1–HRI–ISR axis.

Study	Year	Key finding	Experimental system
Fessler et al. (Nature 579:433)	2020	Identified OMA1–DELE1–HRI pathway relaying mitochondrial stress to cytosol via ISR	Genome-wide CRISPR screens (KBM7)
Guo et al. (Nature 579:427)	2020	Independently identified OMA1–DELE1–HRI; DELE1-S activates HRI	CRISPR screens (HEK293T)
Yang J et al. (Nat Struct Mol Biol 30:1295)	2023	DELE1 oligomerizes into octamers via TPR domains to activate HRI	Cryo-EM, biochemistry
Akabane et al. (Cell Rep 42:112454)	2023	Identified TIM23 in PINK1 complex; TIM23 protects PINK1 from OMA1	Mass spectrometry, HeLa
Eldeeb et al. (PNAS 121:e2313540121)	2024	PINK1-TOM-TIM23 supercomplex; TOMM20 gates PINK1 via NT-CTE	HEK293T, iPSC-DA neurons, organoids
Bi et al. (Commun Biol 7:391)	2024	DELE1-VS identified; HtrA2 as second DELE1 protease; PD G399S impairs cleavage	HEK293T, HeLa
Haakonsen et al. (Nature 626:874)	2024	Discovered SIFI E3 ligase; converging degrons; UBR4 mutations cause neurodegeneration	Biochemistry, human genetics
Singh et al. (Sci Adv 11:eadn2528)	2025	Kinome screen: HRI as negative regulator of PINK1 mitophagy	HeLa, SK-OV-3, U2OS, ARPE-19
Yang M et al. (Nat Commun 17:4838)	2026	ISR suppresses mitophagy by preserving import efficiency via translation (ATF4-independent)	U2OS, HEK293T
Thayer et al. (EMBO J 45:64)	2025	Unified mechanism: Diverse damage converges on MMP loss; TOMM5 required for PINK1	CRISPRi screens, MFN2-Halo
Lorriman et al. (EMBO J 45:4447)	2026	PINK1 TMD structural plasticity governs PARL vs. ROMO1 fate	AlphaFold, MitoLuc assay

**TABLE 3:** Parkinson’s disease-associated mutations and therapeutic targets in the mitochondrial import stress axis.

Gene/Protein	Mutation/Intervention	Effect on axis	PD association/Status
PINK1	G309D, L347P, W437X, *etc.* (PARK6)	Disrupts NT-CTE–TOMM20 interaction; impaired supercomplex	Autosomal recessive early-onset PD
Parkin (PRKN)	R42P, C212Y, T240R, *etc.* (PARK2)	Loss of E3 ligase activity; impaired mitochondrial ubiquitination	Most common cause of AR early-onset PD
HtrA2	G399S, A141S	Reduced DELE1 processing (DELE1-VS)	PD risk factor; essential tremor
UBR4 (SIFI)	Loss-of-function mutations	Persistent DELE1–HRI–ISR; sustained ATF4/CHOP → apoptosis	Ataxia/early-onset dementia
α-Synuclein (SNCA)	A53T, A30P, E46K, duplication/triplication	Binds TOM20; inhibits mitochondrial protein import	Autosomal dominant PD (PARK1/4)
USP30	Pharmacological inhibition	Enhances Parkin-mediated ubiquitination; promotes mitophagy	Preclinical efficacy demonstrated in PD models
eIF2B	ISRIB (activator)	Reverses p-eIF2α effects; enhances PINK1/mitophagy	Preclinical; rodent safety established
HRI (EIF2AK1)	Selective inhibitor (theoretical)	Blocks DELE1–HRI; relieves ISR brake on mitophagy	Preclinical concept
DELE1	Genetic KD/inhibitor (theoretical)	Prevents ISR-mediated mitophagy suppression	Preclinical concept

PINK1, Parkin (PRKN), HtrA2, and α-synuclein (SNCA) are established PD-linked genes; UBR4 (SIFI) loss-of-function is associated with ataxia and early-onset dementia rather than PD., The HRI, inhibitor and DELE1 knockdown are conceptual targets for which no clinical compounds currently exist; USP30 inhibition has shown neuroprotective efficacy in preclinical PD, models, while eIF2B modulation (ISRIB) remains preclinical.

Literature search strategy. This is a narrative review. We searched PubMed and bioRxiv (through June 2026) using combinations of the terms “PINK1”, “Parkin”, “DELE1”, “HRI”, “EIF2AK1”, “integrated stress response”, “SIFI”, “UBR4”, and “mitochondrial protein import”, supplemented by forward- and backward-citation tracking of key primary studies. Studies were prioritized for mechanistic depth, experimental rigor, and relevance to Parkinson’s disease, with emphasis on the 2020–2026 literature. The corresponding mechanistic framework—from mitochondrial import stress sensing and PINK1 fate determination to DELE1–HRI–ISR activation and acute-versus-chronic stress responses—is illustrated in Figures 1–4, respectively.

**FIGURE 1:**
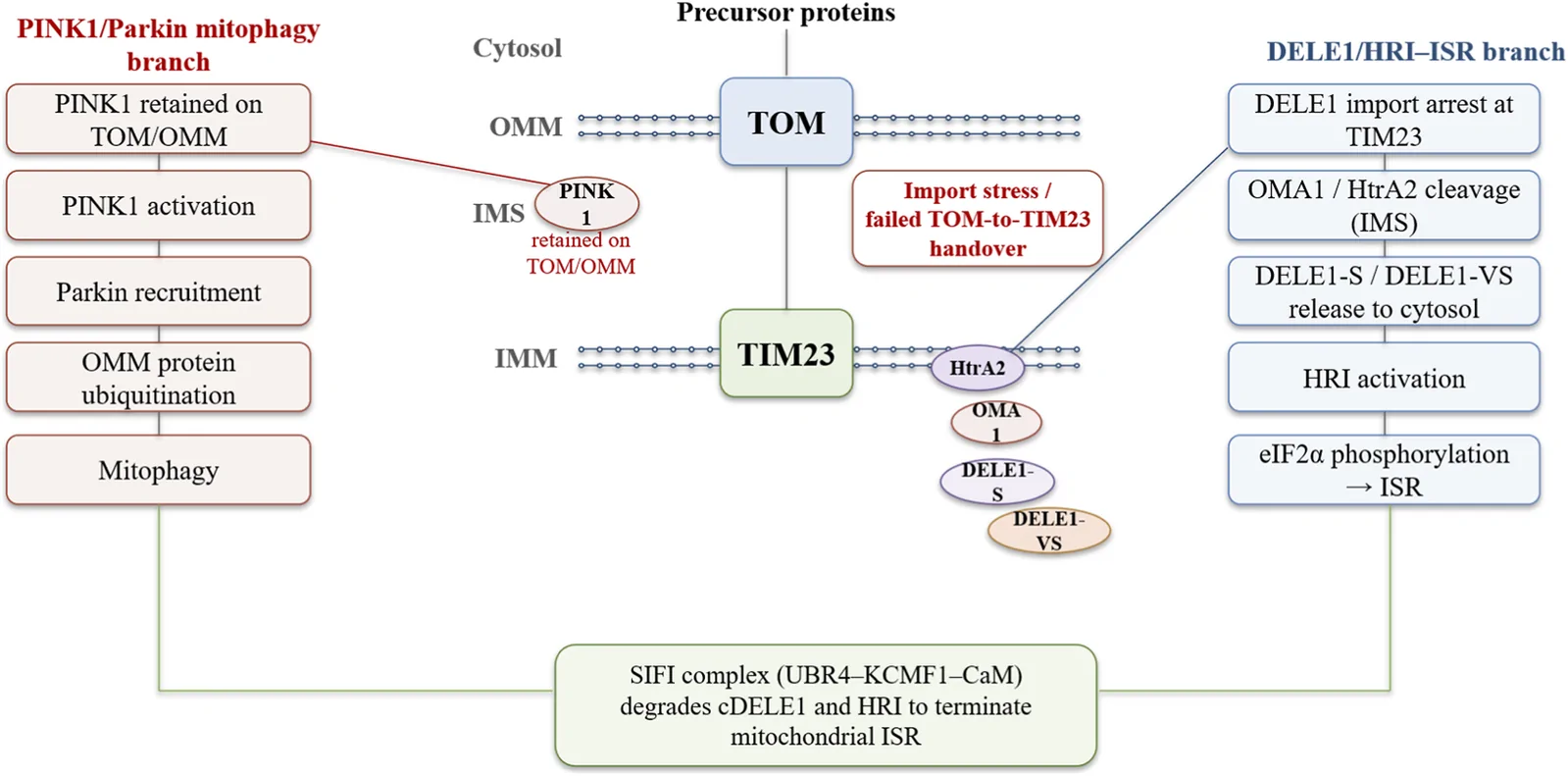
TOM/TIM23 mitochondrial protein import machinery as a stress-sensing hub. Under basal conditions, mitochondrial precursor proteins are imported through the TOM and TIM23 translocases. When the TOM-to-TIM23 handover is disrupted, mitochondrial import stress engages two interconnected quality-control pathways. PINK1 is retained at the outer mitochondrial membrane, where it activates Parkin-dependent ubiquitination and mitophagy. In parallel, impaired DELE1 import promotes OMA1-and HtrA2-dependent processing, generating the DELE1-S and DELE1-VS fragments that are released into the cytosol to activate HRI, eIF2α phosphorylation, and the integrated stress response (ISR). The SIFI complex (UBR4–KCMF1–calmodulin) limits the duration of this response by targeting cleaved DELE1 and HRI for degradation.

**FIGURE 2:**
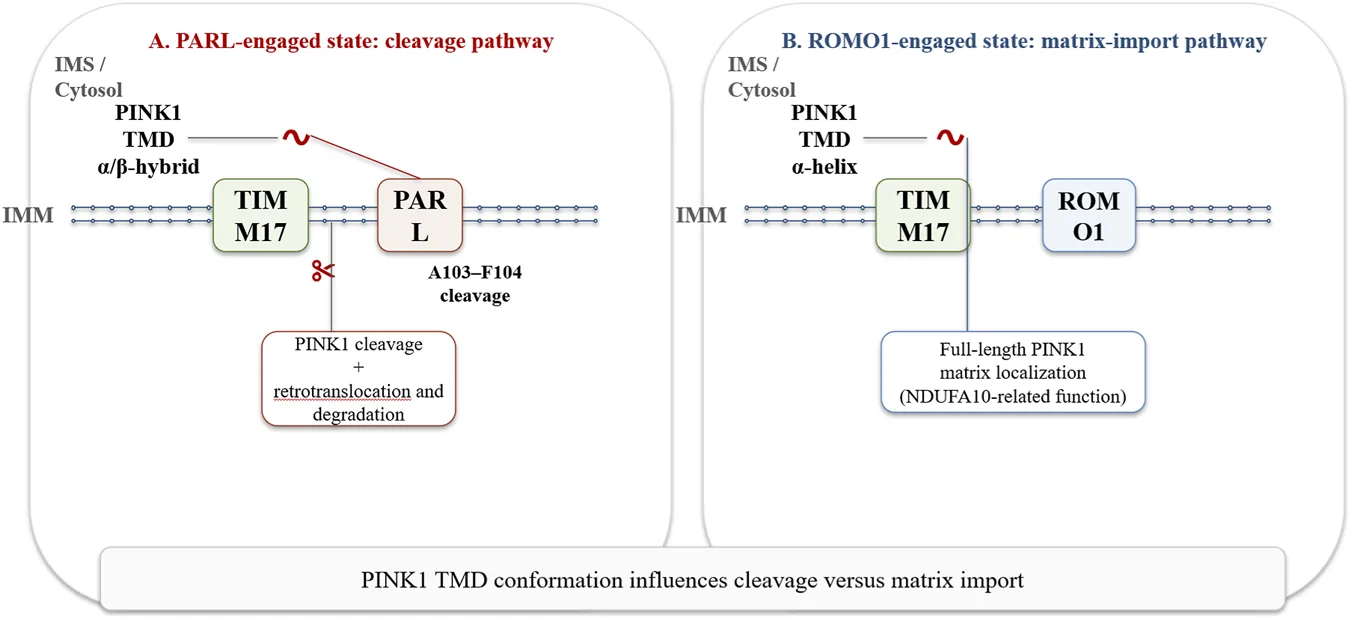
Structural plasticity of PINK1 at the TIM23 interface. The proposed TIM23 branch point links the conformation of the PINK1 transmembrane domain (TMD) to distinct import fates. At the TIMM17–PARL interface, the PINK1 TMD adopts an α/β-hybrid conformation that favors PARL-mediated cleavage at A103–F104, followed by retrotranslocation and degradation. In the alternative TIMM17–ROMO1 state, the TMD remains predominantly α-helical and favors continued translocation of full-length PINK1 into the mitochondrial matrix, where matrix-localized PINK1 has been linked to NDUFA10 phosphorylation. This branch-point model is based on recent structural modeling and cell-based import assays and remains to be independently validated.

**FIGURE 3:**
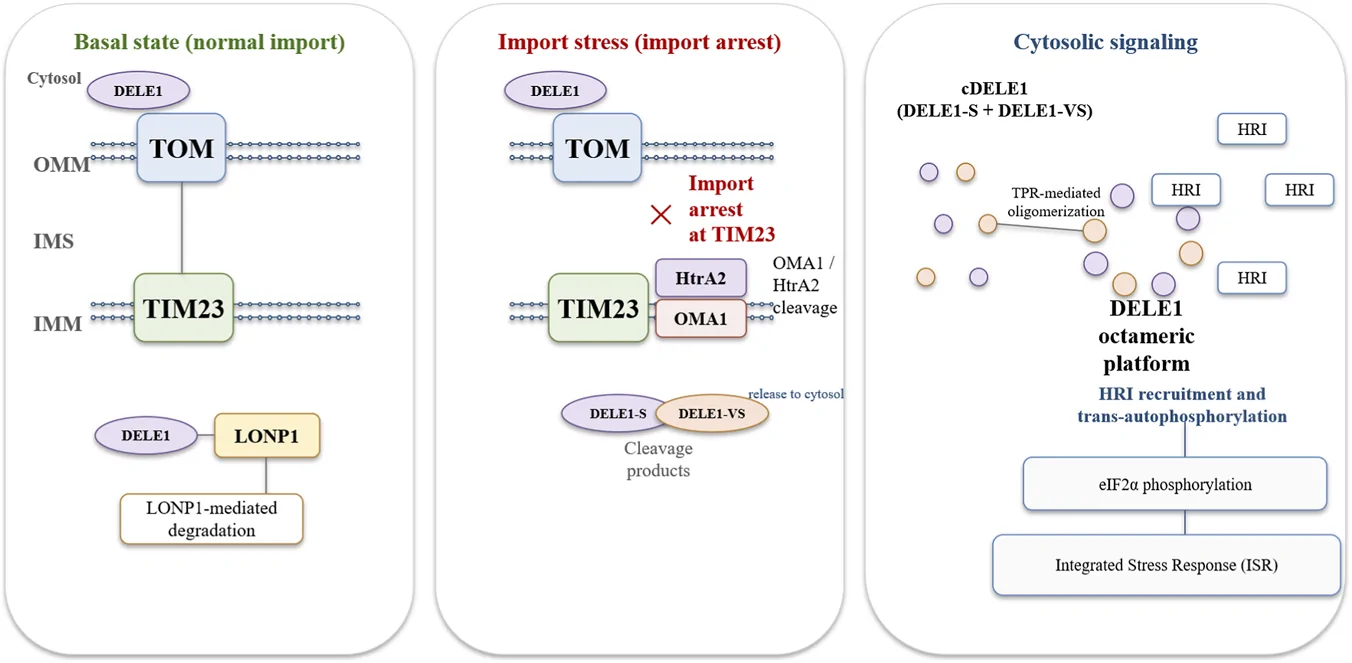
DELE1 processing, oligomerization, and HRI–ISR activation during mitochondrial import stress. Under basal conditions, DELE1 is imported through TOM/TIM23 into the mitochondrial matrix and undergoes LONP1-mediated degradation. During import stress, DELE1 import is arrested and processing by OMA1 and HtrA2 generates the C-terminal fragments DELE1-S and DELE1-VS, which are released into the cytosol. Cleaved DELE1 species (cDELE1) oligomerize through their TPR domains to form an octameric signaling platform that recruits and promotes trans-autophosphorylation of HRI. Activated HRI subsequently phosphorylates eIF2α, leading to induction of the integrated stress response.

**FIGURE 4:**
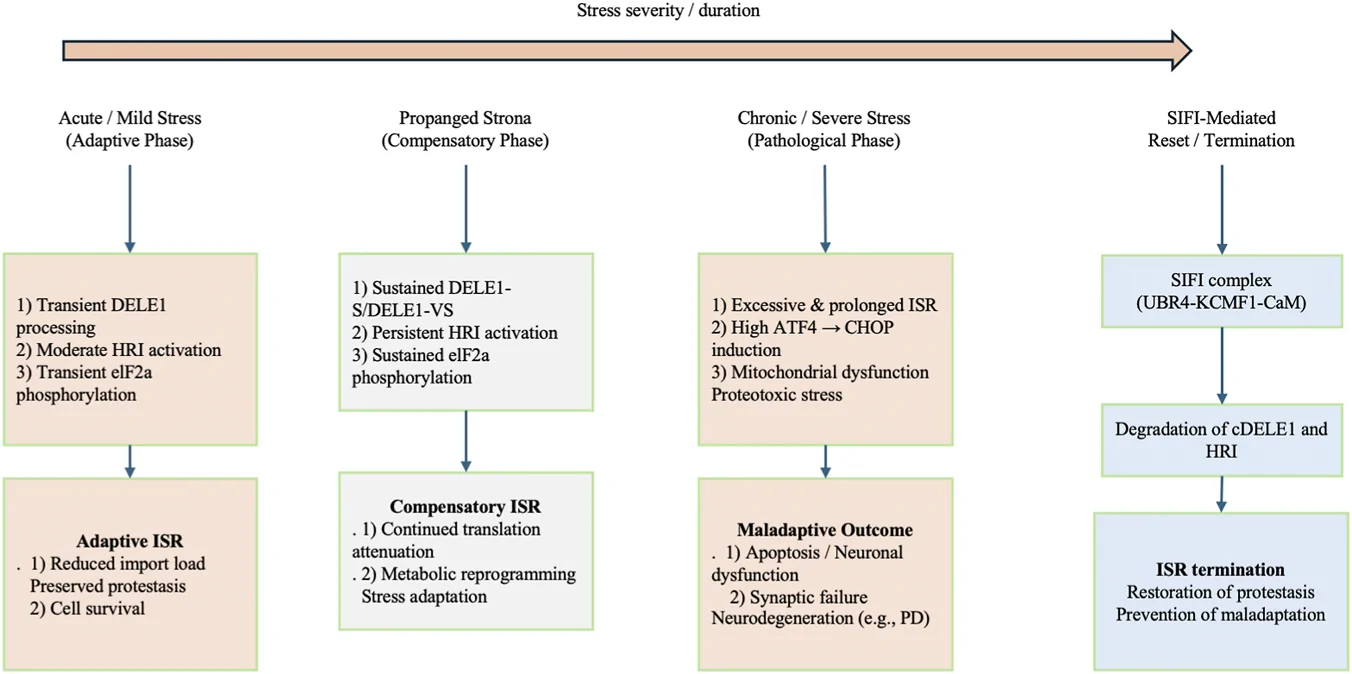
The acute-versus-chronic stress model. Under acute, resolvable stress the DELE1–HRI–ISR axis is protective and SIFI terminates the response; under chronic, unresolvable stress, persistent ISR activation and SIFI saturation drive CHOP-mediated apoptosis.

## 2 The mitochondrial protein import machinery: architecture and stress-sensing function

### 2.1 The TOM complex: universal entry gate

The TOM complex is a multi-subunit assembly in the mitochondrial outer membrane that recognizes, translocates, and sorts virtually all nuclear-encoded mitochondrial precursor proteins (Araiso et al., 2019; Tucker and Park, 2019). Its core is the β-barrel pore protein TOMM40, through which unfolded precursors transit. Receptor subunits handle substrate recognition: TOMM20 binds N-terminal mitochondrial targeting sequences (MTS), TOMM22 provides a docking site for precursors emerging from the TOMM40 channel, and TOMM70 recognizes internal targeting signals, often cooperating with cytosolic Hsp70/Hsp90 chaperones (Backes et al., 2018). Smaller regulatory subunits—TOMM5, TOMM6, and TOMM7—modulate complex stability and substrate handover.

Cryo-EM structures of the TOM holocomplex from yeast and human cells show a dimeric organization, with two TOMM40 pores arranged symmetrically and receptor subunits on the cytosolic face (Araiso et al., 2019; Wang et al., 2020). The complex is not a passive pore; it actively sorts precursors to specific downstream translocases, with different substrates requiring different subunits. This functional specialization matters for PD: TOMM20 and TOMM7 play non-redundant roles in PINK1 stabilization and activation (Eldeeb et al., 2024; Bayne, 2024).

### 2.2 The TIM23 complex: inner membrane translocase and sorting platform

Following passage through the TOM complex, precursors destined for the mitochondrial matrix or inner membrane are engaged by the TIM23 complex. The core translocation channel is formed by TIMM23 and TIMM17, with TIMM50 serving as the primary receptor that captures precursors emerging from the TOM complex (Mokranjac and Neupert, 2010; Callegari et al., 2020). The membrane potential (Δψm) across the inner membrane provides the electrophoretic driving force for the initial translocation of the positively charged MTS through TIM23, while the presequence translocase-associated motor (PAM) complex—comprising mtHsp70, TIMM44, TIMM16 (PAM16), TIMM14 (PAM18), and the nucleotide exchange factor Mge1—completes translocation into the matrix through ATP-dependent pulling (Demisht et al., 2017).

The TIM23 complex also serves as a critical sorting platform. Precursors containing a hydrophobic sorting signal downstream of the MTS are laterally released into the inner membrane via the TIM23-SORT pathway, while matrix-destined precursors are fully translocated. Within the matrix, the mitochondrial processing peptidase (MPP) proteolytically removes the MTS, and for select substrates, the inner membrane rhomboid protease PARL (presenilin-associated rhomboid-like protein) performs an additional intramembrane cleavage (Spinazzi and De Strooper, 2016). Both MPP and PARL are essential for the normal processing of PINK1, and their dysfunction is directly linked to impaired PINK1/Parkin signaling.

### 2.3 Mitochondrial protein import as a stress sensor

The discovery that import competence—rather than membrane potential alone—is the primary trigger for stress pathway activation changed how the field thinks about mitochondrial quality control. PINK1 accumulation was first thought to require Δψm loss, since the protonophore CCCP robustly induces PINK1 stabilization (Narendra et al., 2010). But PINK1 can also accumulate with Δψm intact—for instance, when a misfolded matrix protein (ΔOTC) overwhelms the import machinery (Jin and Youle, 2013), or when TIM23 itself is inhibited (Akabane et al., 2023).

DELE1 cleavage and ISR activation followed a similar story. Initially attributed to OMA1 activation downstream of depolarization, it is now clear that diverse import stresses converge on DELE1 processing: TIM23 knockdown, TRAP1 inhibition, pharmacologic blockade with MitoBlock compounds (Fessler et al., 2020; Guo et al., 2020; Bi et al., 2024). Systematic CRISPRi screens support a unifying model: the TOM-to-TIM23 handover is the critical checkpoint. Anything that disrupts this step—depolarization, translocase inhibition, precursor overcrowding—triggers both PINK1 retention on TOM and DELE1 retention and cleavage (Thayer et al., 2025).

The TOM/TIM23 system is thus not just a transport conduit. It is the central stress-sensing hub of the mitochondrion, translating diverse insults into discrete signals through the PINK1/Parkin and DELE1/HRI branches.

## 3 The PINK1/Parkin branch: sensing import failure to initiate mitophagy

### 3.1 PINK1: a sensor of mitochondrial import competence

PINK1 (PTEN-induced putative kinase 1) is a 581-amino acid serine/threonine kinase that contains an N-terminal MTS, a hydrophobic transmembrane domain (TMD, residues ∼94–110), and a C-terminal kinase domain facing the cytosol (when arrested on the outer membrane) (Nar et al., 2024; Valente et al., 2004). In healthy mitochondria, PINK1 is constitutively imported through TOM and TIM23 in a Δψm-dependent manner. Upon reaching the inner membrane, its TMD is cleaved by PARL between residues Ala103 and Phe104 (Jin et al., 2010; Deas et al., 2011). The cleaved fragment is retro-translocated to the cytosol, where its exposed N-terminal phenylalanine is recognized by the N-degron pathway and targeted for proteasomal degradation (Yamano et al., 2013). This constitutive import-and-degrade cycle ensures that PINK1 protein levels are kept extremely low under basal conditions.

When import is compromised, full-length PINK1 fails to engage TIM23 and instead accumulates as a dimer on the TOM complex, kinase domain facing the cytosol (Okatsu et al., 2015; Rasool et al., 2022). Recent work shows that PINK1 does not simply stall as an isolated molecule: it organizes a ∼720–800 kDa supercomplex containing both TOM and TIM23 subunits (Eldeeb et al., 2024; Akabane et al., 2023), demonstrated largely in cultured cell lines, with additional evidence in iPSC-derived dopaminergic neurons and organoids. The supercomplex depends on PINK1 itself—it physically tethers TOM and TIM23 through direct interactions with the cytosolic domain of TOMM20. The PINK1 NT-CTE module (N-terminal helix and C-terminal extension) mediates this binding, and disease-associated mutations at this interface, including those found in familial PD, disrupt supercomplex formation and downstream mitophagy (Eldeeb et al., 2024).

### 3.2 Parkin activation and the ubiquitin signaling cascade

PINK1 phosphorylates ubiquitin at Ser65 upon stabilization on the mitochondrial surface (Kane et al., 2014; Koyano et al., 2014). This does two things: it recruits the E3 ligase Parkin from the cytosol to mitochondria, and it primes Parkin’s ubiquitin-like (UBL) domain for phosphorylation at the same residue, relieving autoinhibition and triggering catalytic activity (Sauvé et al., 2022) (Gladkova et al., 2018). Activated Parkin then ubiquitinates a broad set of outer membrane proteins—TOMM20, TOMM70, mitofusins (MFN1/2), VDAC, MIRO—generating ubiquitin chains that recruit mitophagy receptors and drive autophagosome engulfment (Sarraf et al., 2013; Ordureau et al., 2018). These conclusions derive largely from immortalized cell lines and cell-free reconstitution assays and have not yet been systematically validated in primary dopaminergic neurons or *in vivo*.

Quantitative proteomics has identified over 100 mitochondrial targets of Parkin (identified in cultured cells). It selectively builds K6-, K11-, K48-, and K63-linked chains that recruit distinct downstream effectors (Ordureau et al., 2014; Swatek et al., 2019). The autophagy receptors OPTN, NDP52, TAX1BP1, NBR1, and p62 are recruited to these chains. OPTN and NDP52 are the primary receptors for autophagosome engagement via their LC3-interacting region (LIR) motifs (Lazarou et al., 2015; Vargas et al., 2019). TANK-binding kinase 1 (TBK1) phosphorylates OPTN and NDP52 to enhance their binding to ubiquitin chains and LC3, and together with NDP52 recruits and activates the autophagy-initiating kinase ULK1 (Unc-51-like autophagy-activating kinase 1), coupling receptor engagement to autophagosome formation (Vargas et al., 2019). In neurons, OPTN appears to play a particularly prominent role; p62 contributes more to aggregate clearance than to mitophagy itself (Evans and Holzbaur, 2020).

### 3.3 TOM subunit requirements and the PINK1 import arrest model

A systematic dissection of TOM subunit requirements for PINK1 stabilization reveals a hierarchy (largely in genome-edited cultured cell lines). TOMM40 and TOMM22 are essential, consistent with their roles as the core pore and primary receptor (Thayer et al., 2025). TOMM7 is also strictly required. TOMM20 plays a regulatory role: it directly binds PINK1 and is needed for full kinase activation, but not for initial PINK1 retention. TOMM70 is largely dispensable for endogenous PINK1, though it may engage excess PINK1 through Hsp70 (Eldeeb et al., 2024).

The requirement for TOMM5 is particularly interesting. TOMM5, a small (∼5 kDa) subunit with a single transmembrane helix, is essential for maintaining PINK1–TOMM40 contacts during import arrest (Thayer et al., 2025). Loss of TOMM5 does not impair general import but specifically blocks PINK1 accumulation in cultured cells, suggesting it creates a dedicated docking site for arrested PINK1 on the TOM complex. This points to functional specialization within the TOM complex: PINK1 has co-evolved with specific TOM subunits to exploit structural features of the import pore.

### 3.4 Beyond depolarization: multiple routes to PINK1 activation

A key advance is the recognition that diverse mitochondrial insults—not just depolarization—can trigger PINK1 accumulation and mitophagy. Expressing ΔOTC, a misfolded variant of ornithine transcarbamylase, induces PINK1 stabilization and Parkin recruitment without detectable Δψm loss (Jin and Youle, 2013). This initially seemed puzzling, but it is now explained by a model in which ΔOTC saturates matrix chaperone and protease capacity, indirectly impairing the TIM23 motor (PAM complex) and creating precursor “traffic jams” that extend back to the TOM-TIM23 interface. Similarly, inhibiting the matrix protease LONP1 triggers PINK1-dependent mitophagy by allowing import-incompetent substrates to accumulate and block the translocase (Bota and Davies, 2016). Together, these observations show that PINK1 functions as a general sensor of import competence—more precisely, of the efficiency of precursor transfer from TOM to TIM23—rather than a specific sensor of membrane potential. This conclusion rests on experiments in cultured cell lines and awaits validation in primary neurons and *in vivo*.

### 3.5 Structural plasticity of PINK1 at the TIM23 interface

Recent structural modeling and biochemical analysis have suggested an unexpected layer of complexity in PINK1 import: the TMD of PINK1 can adopt alternative conformations at the TIM23 interface, determining whether the precursor is fully imported and cleaved by PARL or diverted into the matrix (Lorriman et al., 2026). This branch-point model currently rests on a single study combining AlphaFold-based structural modeling with cell-based import assays (Lorriman et al., 2026) and awaits independent validation in intact cells and neurons. When the TMD forms an α/β-hybrid structure at the TIMM17–PARL interface, it retracts into PARL’s active site for cleavage between A103–F104, initiating the canonical degradation pathway. In contrast, when the TMD remains an α-helix at the TIMM17–ROMO1 interface, PINK1 is fully translocated into the matrix—a fate that may be physiologically significant, as matrix-localized full-length PINK1 has been reported to phosphorylate NDUFA10, a subunit of Complex I (Morais et al., 2014).

The structural plasticity of PINK1’s TMD and the mutually exclusive nature of PARL versus ROMO1 engagement with TIM23 raise the possibility of a regulated branch point that could fine-tune the balance between PINK1 degradation, mitophagy initiation, and matrix functions. The molecular determinants governing this choice remain poorly understood but likely involve the relative occupancy of TIMM17 by PARL versus ROMO1, which may be influenced by metabolic state, redox conditions, and the overall rate of protein import.

## 4 The DELE1/HRI branch: coupling import stress to the integrated stress response

### 4.1 DELE1: a mitochondrial stress sentinel

DELE1 (DAP3-binding cell death enhancer 1) is a 517-amino acid protein that, like PINK1, is constitutively imported into the matrix via TOM-TIM23 and rapidly degraded by LONP1 under basal conditions (Fessler et al., 2020; Guo et al., 2020) (demonstrated in cultured cells). It contains two tandem tetratricopeptide repeat (TPR) domains that mediate protein interactions; these TPR domains within the C-terminal fragment are responsible for binding and activating HRI.

The discovery of DELE1 as the long-sought mitochondrial ISR signal was reported independently by two groups in 2020 using genome-wide CRISPR screens (Fessler et al., 2020; Guo et al., 2020). When import is impaired, DELE1 import stalls, exposing it to proteolysis. The OMA1 metalloprotease, activated by mitochondrial stress, cleaves DELE1 near His142, generating a ∼40 kDa C-terminal fragment (DELE1-S) that is released into the cytosol, as demonstrated in cultured cell lines (Guo et al., 2020). DELE1-S oligomerizes into octamers through its TPR domains, forming a platform that recruits and activates HRI via trans-autophosphorylation (Yang et al., 2023).

### 4.2 Two forms of DELE1: DELE1-S and DELE1-VS

Bi and colleagues (Bi et al., 2024) identified a second, shorter cleavage product, DELE1-VS (∼37 kDa), generated by cleavage within residues 456–466 near the C-terminus of DELE1-S. DELE1-VS accumulates preferentially under non-depolarizing import stress—oligomycin, mitoblock compounds—and retains the TPR domains required for HRI binding. It is fully competent to activate the ISR in cell-based assays.

Bi et al. (Bi et al., 2024) also showed that OMA1 is not the only DELE1 protease. OMA1 was required for DELE1 cleavage in HeLa cells but dispensable in HEK293T cells, pointing to cell-type-specific mechanisms. The mitochondrial serine protease HtrA2, which resides in the intermembrane space, was identified as a second DELE1 protease that specifically generates DELE1-VS. HtrA2 is directly relevant to PD: loss-of-function mutations in *HTRA2* are linked to PD and essential tremor (Strauss et al., 2005; Bogaerts et al., 2008), and the PD-associated G399S HtrA2 mutant shows reduced DELE1 processing activity in overexpression systems (Bi et al., 2024). This establishes a mechanistic link between HtrA2, DELE1 signaling, and PD that is independent of HtrA2’s canonical role in apoptosis.

### 4.3 HRI kinase: the mitochondrial ISR effector

HRI (heme-regulated inhibitor, EIF2AK1) is one of four mammalian eIF2α kinases, alongside PKR (EIF2AK2), PERK (EIF2AK3), and GCN2 (EIF2AK4). HRI was originally characterized as a heme-sensitive regulator of globin synthesis in erythroid cells, but the discovery that it is the primary kinase activated by mitochondrial stress—through its direct binding to DELE1-S oligomers—has redefined its functional significance (Guo et al., 2020; Yang et al., 2023). This was demonstrated in cultured cell lines and *in vitro* reconstitution systems.

Upon activation, HRI phosphorylates the α subunit of eukaryotic initiation factor 2 (eIF2α) at Ser51. Phosphorylated eIF2α acts as a competitive inhibitor of the guanine nucleotide exchange factor eIF2B, reducing global translation initiation while paradoxically enhancing the translation of select mRNAs containing upstream open reading frames (uORFs), most notably the transcription factor ATF4 (activating transcription factor 4) (Pakos-Zebrucka et al., 2016; Costa-Mattioli and Walter, 2020). ATF4 in turn drives the expression of a transcriptional program that includes genes involved in amino acid metabolism, antioxidant defense, and the pro-apoptotic factor CHOP (DDIT3). The ISR thus orchestrates a dual response: a rapid translational brake that reduces the burden of newly synthesized proteins entering mitochondria, and a slower transcriptional reprogramming that reconfigures cellular metabolism. This ISR-mediated adaptive program is particularly relevant to the nervous system, where ATF4-dependent metabolic rewiring promotes neural stem cell survival under conditions of mitochondrial dysfunction (Iqbal et al., 2024).

### 4.4 DELE1-independent ISR activation via iron deficiency

An additional layer of complexity in the DELE1–HRI axis was revealed by the finding that iron deficiency can activate HRI through an OMA1-independent, DELE1-dependent mechanism (Sekine et al., 2023). Under iron-limited conditions, DELE1 import into the mitochondrial matrix is impaired independently of Δψm, causing full-length DELE1-L to accumulate on the outer mitochondrial membrane. From this location, DELE1-L can interact with and activate HRI without prior cleavage, establishing a mode of ISR activation that bypasses OMA1 entirely (in cultured cells). This finding has potential relevance to PD, as iron accumulation in the substantia nigra is a well-established feature of the disease (Dexter et al., 1991; Mochizuki et al., 2020), and iron dyshomeostasis may differentially modulate DELE1–HRI signaling in dopaminergic neurons.

## 5 The ISR as a negative regulator of PINK1/Parkin mitophagy

### 5.1 Identification of HRI as a mitophagy brake

The crosstalk between these two branches came into focus through a whole-human-kinome siRNA screen by Singh, Agarwal, Volpi, and colleagues in the Muqit laboratory (Singh et al., 2025). Screening for modifiers of PINK1 stabilization in response to mitochondrial stress (oligomycin/antimycin A), they identified HRI (EIF2AK1) as a top hit: HRI knockdown strongly enhanced PINK1 protein levels, ubiquitin Ser65 phosphorylation, and phosphorylation of the downstream Parkin substrate Rab8A at Ser111.

The effect was specific to HRI. Knockdown of the other three eIF2α kinases—PKR, PERK, GCN2—had no effect on PINK1 stabilization. DELE1 knockdown produced the same enhancement, confirming that the DELE1–HRI–eIF2α phosphorylation axis, not a moonlighting function of HRI, was responsible. The findings held across multiple human cell lines (HeLa, SK-OV-3, U2OS, ARPE-19) and were confirmed in CRISPR *EIF2AK1* knockout clones (Singh et al., 2025).

### 5.2 Mechanism: translational control of mitochondrial import efficiency

Two complementary studies elucidated the mechanism. Singh et al. (Singh et al., 2025) showed that HRI knockout or DELE1 knockdown caused both transcriptional upregulation of *PINK1* mRNA (blocked by DRB and α-amanitin) and enhanced PINK1 translation (blocked by cycloheximide). The ISR thus limits PINK1 expression at both the transcriptional and translational levels.

Yang and colleagues (Yang et al., 2026) provided a mechanistic explanation rooted in import dynamics. Under non-depolarizing stress (oligomycin), the ISR preserves mitochondrial import efficiency by reducing the flux of newly synthesized proteins entering the import pathway. When the ISR is intact, translation attenuation eases the burden on the import machinery, leaving enough residual capacity to maintain PINK1 import and degradation—preventing mitophagy. When the ISR is abrogated (DELE1 knockdown or ISRIB), unchecked protein synthesis overwhelms the import machinery. PINK1 import fails, PINK1 accumulates, and mitophagy is triggered—even with Δψm preserved.

Yang and colleagues (Yang et al., 2026) showed that this import-preserving function does not require ATF4-mediated transcription. Low-dose cycloheximide in ISR-deficient cells was enough to restore import efficiency and suppress PINK1 accumulation. Translational control—the rapid, ATF4-independent arm of the ISR—is the primary mechanism by which DELE1–HRI restrains mitophagy.

### 5.3 ISRIB: pharmacological proof of principle

The small molecule ISRIB (integrated stress response inhibitor) has been a useful tool for dissecting the ISR–mitophagy crosstalk. ISRIB binds and stabilizes the eIF2B decamer, rendering it insensitive to inhibition by phosphorylated eIF2α and thereby reversing the translational consequences of ISR activation (Sidrauski et al., 2015; Tsai et al., 2018). Treatment with ISRIB phenocopied the effects of HRI or DELE1 knockdown, enhancing PINK1 stabilization and activation in response to mitochondrial stress (Singh et al., 2025; Yang et al., 2026). This pharmacological result has two important implications: first, it confirms that the eIF2α phosphorylation step is the critical node at which the ISR suppresses mitophagy; second, it identifies ISRIB—and potentially other ISR-modulating compounds—as candidate agents for enhancing PINK1/Parkin-dependent mitophagy in PD, building on ISRIB’s established safety profile in preclinical studies.

## 6 The SIFI complex: silencing the mitochondrial ISR

### 6.1 Discovery and composition of the SIFI E3 ligase

The mitochondrial ISR, once activated, must be terminated to prevent sustained translational suppression and eventual CHOP-mediated apoptosis. The SIFI (silencing factor of the integrated stress response) complex, identified by Haakonsen and colleagues (Haakonsen et al., 2024), is a large E3 ubiquitin ligase that terminates the DELE1–HRI signaling arm. SIFI is composed of the E3 ligase UBR4, the adaptor protein KCMF1, and calmodulin. It recognizes both cleaved DELE1 fragments (DELE1-S and DELE1-VS, collectively referred to as cDELE1) and HRI as substrates and targets them for ubiquitin-dependent proteasomal degradation.

SIFI recognizes a class of bifunctional helical motifs—“converging degrons”—shared by mitochondrial presequences, DELE1, and HRI. These degrons encode both localization information (directing proteins to mitochondria) and stability information (marking them as SIFI substrates). Under basal conditions, most mitochondrial precursors are rapidly imported and thus physically sequestered from SIFI in the cytosol. When import stress causes precursors to accumulate in the cytosol, they compete with DELE1 and HRI for SIFI binding, delaying ISR silencing until import recovers and the precursor backlog clears (Haakonsen et al., 2024).

### 6.2 SIFI dysfunction and neurodegeneration

Loss-of-function mutations in *UBR4* cause a progressive neurological disorder with ataxia and early-onset dementia (Haakonsen et al., 2024). In cellular models, UBR4 deficiency causes uncontrolled accumulation of cDELE1 and HRI, persistent eIF2α phosphorylation, sustained ATF4 expression, and neuronal apoptosis. ISRIB treatment rescued the viability of UBR4-deficient neurons: unrestrained ISR signaling, not a pleiotropic effect of UBR4 loss, drives cell death.

For PD, this is instructive. It shows, in a human genetic context, that failure to silence the mitochondrial ISR is sufficient to cause neurodegeneration. *UBR4* mutations have not been directly linked to PD, but the principle—ISR homeostasis is essential for neuronal survival, and its disruption causes cell-type-specific neurodegeneration—may extend to dopaminergic neuron vulnerability. The SIFI discovery also explains mechanistically how the ISR transitions from protective to pathological: when SIFI-mediated silencing is impaired, or when import stress outlasts SIFI’s capacity to clear DELE1 and HRI, the ISR persists and triggers apoptosis through CHOP and related effectors.

## 7 Crosstalk architecture: an integrated model of the TOM/TIM–PINK1/Parkin–DELE1/HRI axis

### 7.1 A unified signaling hub

The convergence of structural, biochemical, genetic, and pharmacological evidence now supports an integrated model in which the TOM/TIM23 translocase system functions as a signaling hub that partitions mitochondrial stress into two interconnected responses. The current model can be summarized as follows:

Under basal conditions, both PINK1 and DELE1 are constitutively imported through TOM and TIM23 and rapidly degraded, keeping both sensors at low steady-state levels (Sections 3.1 and 4.1).

Upon import stress, the TOM-to-TIM23 handover is compromised, engaging both branches in parallel: PINK1 accumulates on the TOM complex and, through Parkin, initiates mitophagy (Section 3), while OMA1-and HtrA2-generated C-terminal fragments (DELE1-S and DELE1-VS) activate HRI and the ISR (Section 4).

The integrated model centers on the timing and competition between these two responses. Translational attenuation driven by the ISR reduces the influx of newly synthesized precursors, preserving residual import capacity and thereby limiting further PINK1 accumulation and mitophagy—a negative-feedback loop that positions the DELE1–HRI–ISR axis as a molecular brake on organelle clearance (Section 5).

The SIFI (UBR4–KCMF1–calmodulin) E3 ligase closes the loop: it recognizes cDELE1 and HRI through converging degrons, and unimported precursors compete for SIFI binding, delaying ISR silencing until import recovers (Section 6).

### 7.2 The timing hypothesis: acute versus chronic stress

The functional relationship between the two branches is best understood through a temporal lens. Under acute, resolvable stress, the DELE1–HRI–ISR axis is protective: translational attenuation reduces the import burden, giving mitochondria time to recover while avoiding wholesale mitophagy. SIFI then silences the ISR once import is restored. The ISR acts as a transient brake, buying time for repair.

Under chronic, unresolvable stress—as may occur in aging dopaminergic neurons with accumulated mitochondrial damage—persistent ISR activation and progressive SIFI saturation drive sustained translational suppression, chronic ATF4 expression, and CHOP-mediated apoptosis. At the same time, ISR-mediated mitophagy suppression prevents clearance of damaged mitochondria. Dysfunctional organelles accumulate, generating ROS and releasing pro-apoptotic factors. This dual failure—impaired mitophagy plus unresolved ISR signaling—may be a central pathogenic mechanism in P.

### 7.3 Cell-type-specific considerations

Dopaminergic neurons of the substantia nigra possess several features that may render them particularly vulnerable to disruptions in the TOM/TIM–PINK1/Parkin–DELE1/HRI axis. First, these neurons maintain extraordinarily extensive axonal arbors, with individual substantia nigra dopaminergic neurons estimated, on the basis of rodent tracing studies, to support over 200,000 synaptic terminals (Matsuda et al., 2009). The bioenergetic demands of maintaining ion gradients and synaptic vesicle cycling across this vast arbor require a correspondingly large and widely distributed mitochondrial network, placing sustained pressure on mitochondrial import, quality control, and turnover mechanisms.

Second, dopaminergic neurons are autonomous pacemakers that rely on L-type voltage-gated calcium channels (Ca_v1.3) for rhythmic activity, resulting in sustained cytosolic calcium oscillations that impose additional oxidative stress on mitochondria (Guzman et al., 2010; Surmeier et al., 2017). Third, the metabolism of dopamine itself generates reactive quinones and oxidative species that can directly damage mitochondrial proteins and impair import competence (Burbulla et al., 2017). Fourth, recent work has revealed a PINK1-independent role for Parkin in dopaminergic neurons, where CaMKII-mediated Parkin activation promotes synaptic vesicle endocytosis through synaptojanin-1 binding (Song and Krainc, 2024). Loss of Parkin function in this context leads to defective synaptic vesicle recycling and accumulation of toxic oxidized dopamine—a mechanism that may contribute to selective vulnerability independently of mitophagy defects.

Collectively, these features suggest that dopaminergic neurons operate at a narrow margin of mitochondrial proteostatic reserve, such that even modest impairments in import efficiency, DELE1 processing (as with HtrA2 G399S), SIFI function, or ISR dynamics could push the system from compensated stress toward decompensation and neurodegeneration.

## 8 Therapeutic implications

### 8.1 Enhancing PINK1/Parkin mitophagy through ISR inhibition

The finding that ISR inhibition—genetic (DELE1 or HRI knockdown) or pharmacological (ISRIB)—enhances PINK1/Parkin-dependent mitophagy opens a potential therapeutic avenue for PD. ISRIB has an acceptable safety profile in preclinical rodent studies (Costa-Mattioli and Walter, 2020). But clinical translation of ISR inhibition is not straightforward. The ISR is a broadly deployed stress response; systemic inhibition could impair adaptation in tissues facing proteotoxic, metabolic, or infectious stress. DNL343 has demonstrated CNS penetration, ISR target engagement, and early clinical tolerability in individuals with ALS (Flores et al., 2025), while fosigotifator (ABBV-CLS-7262) has been developed as a potent eIF2B activator with pharmacological properties suitable for clinical investigation (Frost et al., 2026). However, whether eIF2B modulation can provide meaningful disease-modifying efficacy in neurodegenerative disorders remains uncertain. Moreover, chronic ISR suppression itself may be a double-edged sword: because the ISR is a broadly protective homeostatic program, sustained blockade risks impairing cellular adaptation and promoting rather than preventing neurodegeneration. For example, in dopaminergic neurons—which already operate at a narrow margin of proteostatic reserve—chronically dampening this adaptive response could reduce their capacity to cope with unrelated proteotoxic or infectious challenges, potentially offsetting the benefit of enhanced mitophagy.

A more selective approach would target the mitochondrial ISR specifically—DELE1 or HRI—rather than eIF2α phosphorylation broadly. The DELE1–HRI interaction is specific: other eIF2α kinases do not regulate PINK1 signaling. Selective HRI kinase inhibitors could therefore enhance mitophagy in dopaminergic neurons without globally suppressing the ISR (Singh et al., 2025). However, HRI and DELE1 also mediate stress responses beyond mitochondria, and selective, brain-penetrant inhibitors of either target have yet to be developed, so the therapeutic window for this strategy remains undefined. Structural characterization of the DELE1–HRI complex would aid the development of such inhibitors.

### 8.2 USP30 inhibition and the mitophagy pathway

A parallel and more clinically advanced therapeutic strategy targets the mitophagy pathway directly through inhibition of USP30, a mitochondrial deubiquitylase that opposes Parkin-mediated ubiquitination (Bingol et al., 2014; Cunningham et al., 2015). USP30 removes ubiquitin chains from damaged mitochondria, and its inhibition enhances mitophagy in cellular and *in vivo* models of mitochondrial dysfunction. Genetic deletion or pharmacological inhibition of USP30 enhances mitophagy and protects dopaminergic neurons in preclinical models of Parkinson’s disease (Fang et al., 2023), supporting USP30 as a potential disease-modifying target.

The relationship between ISR inhibition and USP30 inhibition as therapeutic strategies is not yet clear. Since ISR activation suppresses mitophagy, ISR inhibition may synergize with USP30 inhibition by simultaneously relieving the ISR brake on mitophagy (ISR-targeted) and enhancing the ubiquitin signal that drives it (USP30-targeted). Alternatively, the two approaches may be effective in different patient populations or disease stages, depending on whether the dominant defect lies in mitophagy initiation (PINK1/Parkin loss-of-function) or mitophagy suppression (ISR hyperactivation).

### 8.3 α-Synuclein and the TOM complex

α-Synuclein, the major constituent of Lewy bodies and a central player in PD pathogenesis, has been shown to interact with the TOM complex and interfere with mitochondrial protein import (Di et al., 2016; Dash and Mahalakshmi, 2025). α-Synuclein misimport through the TOM pore disrupts calcium homeostasis, reduces ATP production, and increases reactive oxygen species generation. This raises the possibility that α-synuclein pathology and mitochondrial import stress are mutually reinforcing: α-synuclein oligomers impair TOM function, triggering import stress that activates both the PINK1/Parkin and DELE1/HRI pathways, while import stress may in turn promote α-synuclein aggregation through oxidative modification or impaired degradation.

Targeting the TOM–α-synuclein interaction, or enhancing TOM complex function and fidelity, could represent a complementary approach to modulating mitochondrial import stress in PD. Small molecules that stabilize TOM complex assembly or promote the handover of precursors from TOM to TIM23 might simultaneously reduce PINK1-dependent mitophagy stress and DELE1-dependent ISR activation.

### 8.4 Restoring SIFI function

The discovery that loss of SIFI function causes neurodegeneration through unrestrained ISR activation suggests that enhancing SIFI-mediated silencing of the mitochondrial ISR could be neuroprotective. Conceptually, this could be achieved by promoting UBR4 expression or activity, enhancing KCMF1 adaptor function, or reducing the competition between unimported mitochondrial precursors and DELE1/HRI for SIFI binding. However, the molecular pharmacology of large E3 ligase complexes is in its infancy, and significant work is needed to identify tractable approaches to SIFI modulation.

## 9 Discussion and future directions

Despite the rapid progress achieved over the past 5 years, several fundamental aspects of the TOM/TIM–PINK1/Parkin–DELE1/HRI axis remain unresolved.

It is worth distinguishing established findings from emerging or speculative models. That PINK1 stabilization and DELE1 processing both signal mitochondrial import failure is now well established across multiple experimental systems. By contrast, the PINK1–TOM–TIM23 supercomplex, SIFI-mediated regulation of the mitochondrial ISR, and the acute-versus-chronic stress model proposed here are promising but remain to be fully validated in physiologically relevant models of PD; they should be regarded as hypotheses rather than settled mechanisms.

A major priority is to clarify the molecular mechanisms that determine the allocation of mitochondrial import stress between the PINK1/Parkin and DELE1/HRI branches. Although both PINK1 and DELE1 respond to import failure at the TOM–TIM23 interface, they initiate distinct and potentially antagonistic outcomes, namely, mitophagy and integrated stress response (ISR) activation. This pathway selection may depend on the nature, duration, and severity of the import defect, cell-type-specific expression of TOM complex subunits, the relative availability of OMA1 and HtrA2 proteases, and the binding affinities of PINK1 and DELE1 for stalled import machinery. Defining these determinants will be essential for understanding how mitochondrial quality-control responses are coordinated under different stress conditions.

The molecular transition from an initially adaptive ISR to chronic stress-induced apoptosis also requires further investigation in dopaminergic neurons. Acute ISR activation can promote cellular adaptation and survival, whereas sustained signaling may induce CHOP-dependent apoptosis and neuronal loss. Potential mechanisms include saturation of stress-induced mitochondrial hyperfusion, accumulation of CHOP beyond a critical threshold, and concurrent activation of additional cell-death pathways. Given the lifelong mitochondrial demands placed on dopaminergic neurons, elucidating the temporal dynamics and regulatory checkpoints governing this transition will be particularly important for Parkinson’s disease pathogenesis.

Further *in vivo* characterization of the PINK1–TOM–TIM23 supercomplex is also needed. The approximately 720–800 kDa PINK1-containing complex has been described mainly in cultured cell lines, and its existence, molecular composition, and functional relevance in adult dopaminergic neurons remain insufficiently established. Future studies should determine whether this supercomplex forms transiently during mitochondrial import arrest, how its assembly is regulated, and whether it disassembles after restoration of protein import competence.

The effects of Parkinson’s disease-associated HtrA2 mutations on endogenous DELE1 processing should be examined in physiologically relevant models. The G399S HtrA2 variant has been reported to impair DELE1 cleavage, suggesting that defective mitochondrial ISR signaling may contribute to disease pathogenesis. However, current evidence is largely derived from overexpression systems. Patient-derived induced pluripotent stem cell neurons, gene-edited isogenic models, and *in vivo* systems will be required to establish how HtrA2 mutations influence DELE1 processing, ISR kinetics, mitochondrial homeostasis, and neuronal survival.

A further translational gap is the relative paucity of direct evidence linking this axis to human Parkinson’s disease. PINK1 and Parkin mutations cause familial parkinsonism, and mitochondrial import defects have been documented in PD, most directly through α-synuclein binding to TOM20 in *postmortem* patient tissue (Di et al., 2016). Mitochondrial dysfunction is likewise well characterized in patient-derived iPSC dopaminergic neurons. However, whether DELE1/HRI signaling, DELE1 processing, or SIFI function is itself dysregulated in PD patients has not yet been directly demonstrated; resolving this through patient-derived iPSC models, *postmortem* tissue, and functional biomarkers is an important priority.

Therapeutic targeting of the DELE1–HRI–ISR pathway will require sufficient selectivity to preserve physiological stress adaptation. Because the ISR participates broadly in immunity, metabolism, proteostasis, and cellular defense, systemic inhibition may produce substantial adverse effects. Structural and functional characterization of the DELE1–HRI interaction may support the development of pathway-selective modulators that reduce maladaptive ISR activation or enhance mitophagy in dopaminergic neurons while preserving signaling mediated by other eIF2α kinases.

Finally, the contribution of mitochondrial import stress in glial cells warrants greater attention. Astrocytes and microglia are important regulators of neuronal metabolism, mitochondrial homeostasis, and neuroinflammation in Parkinson’s disease. Mitochondrial dysfunction in astrocytes may compromise metabolic support and neuroprotective functions, whereas persistent mitochondrial stress in microglia may amplify inflammatory signaling. Defining how the TOM/TIM–PINK1/Parkin–DELE1/HRI axis operates across neuronal and glial populations will provide a more comprehensive understanding of cell-autonomous and non-cell-autonomous mechanisms underlying neurodegeneration.

## 10 Conclusion

The TOM/TIM23 protein import machinery, once viewed simply as a conduit for precursor proteins, is now understood as a stress-sensing hub that partitions mitochondrial insults into two signaling pathways: PINK1/Parkin-mediated mitophagy and DELE1/HRI-mediated ISR activation. These pathways engage in antagonistic crosstalk: the ISR acts as a brake on mitophagy. The cell, at least in the short term, prioritizes mitochondrial repair and translational adaptation over wholesale organelle clearance.

For dopaminergic neurons—which face decades of sustained bioenergetic demand—the balance maintained by this axis may be especially fragile. Genetic evidence implicates multiple components of the system in PD: PINK1 and Parkin loss-of-function cause early-onset parkinsonism, HtrA2 mutations impair DELE1 processing, and SIFI dysfunction causes neurodegeneration through unrestrained ISR signaling. The convergence of these genetic findings on a common axis—mitochondrial import stress sensing and response—argues that dysregulation of this system is a central pathogenic mechanism in PD.

The therapeutic challenge is to translate these insights into disease-modifying treatments. Enhancing PINK1/Parkin mitophagy through ISR inhibition, restoring DELE1/HRI silencing, and protecting the TOM/TIM23 import machinery from α-synuclein-mediated dysfunction are complementary strategies targeting different nodes of the same pathway. The difficulty—and the opportunity—lies in doing this while preserving the physiological stress responses that keep neurons alive.

## Glossary

ALS: amyotrophic lateral sclerosis
AR: autosomal recessive
ATF4: activating transcription factor 4
ATP: adenosine triphosphate
CaM: calmodulin
CaMKII: calcium/calmodulin-dependent protein kinase II
CaV1.3: voltage-gated L-type calcium channel subtype 1.3
CCCP: carbonyl cyanide m-chlorophenyl hydrazone
cDELE1: cleaved DELE1
CHOP: C/EBP homologous protein
CRISPR: clustered regularly interspaced short palindromic repeats
CRISPRi: CRISPR interference
DDIT3: DNA damage-inducible transcript 3
DELE1: DAP3-binding cell death enhancer 1
DELE1-L: full-length DELE1
DELE1-S: short C-terminal fragment of DELE1
DELE1-VS: very-short C-terminal fragment of DELE1
DRB: 5,6-dichloro-1-β-D-ribofuranosylbenzimidazole
eIF2α: eukaryotic translation initiation factor 2 alpha
eIF2B: eukaryotic translation initiation factor 2B
EIF2AK1: eukaryotic translation initiation factor 2 alpha kinase 1
GCN2: general control nonderepressible 2
HRI: heme-regulated inhibitor
Hsp70: heat shock protein 70
Hsp90: heat shock protein 90
HtrA2: high-temperature requirement protein A2
IMM: inner mitochondrial membrane
IMS: mitochondrial intermembrane space
iPSC: induced pluripotent stem cell
iPSC-DA: induced pluripotent stem cell-derived dopaminergic neuron
ISR: integrated stress response
ISRIB: integrated stress response inhibitor
KCMF1: potassium channel modulatory factor 1
KD: knockdown
LC3: microtubule-associated protein 1 light chain 3
LIR: LC3-interacting region
LONP1: Lon peptidase 1, mitochondrial
MFN1/2: mitofusin 1/2
MIRO: mitochondrial Rho GTPase
MPP: mitochondrial processing peptidase
MTS: mitochondrial targeting sequence
NBR1: neighbor of BRCA1 gene 1
NDP52: nuclear dot protein 52 kDa
NDUFA10: NADH:ubiquinone oxidoreductase subunit A10
NT-CTE: N-terminal helix–C-terminal extension module
OMA1: OMA1 zinc metallopeptidase
OMM: outer mitochondrial membrane
OPTN: optineurin
PAM: presequence translocase-associated motor
PARL: presenilin-associated rhomboid-like protein
PD: Parkinson’s disease
PERK: protein kinase R-like endoplasmic reticulum kinase
PINK1: PTEN-induced putative kinase 1
PKR: protein kinase R
PRKN: parkin RBR E3 ubiquitin protein ligase gene
p-eIF2α: phosphorylated eukaryotic translation initiation factor 2 alpha
p62/SQSTM1: sequestosome 1
ROMO1: reactive oxygen species modulator 1
ROS: reactive oxygen species
SIFI: silencing factor of the integrated stress response
siRNA: small interfering RNA
SNCA: alpha-synuclein gene
TAX1BP1: Tax1-binding protein 1
TBK1: TANK-binding kinase 1
TIM: translocase of the inner mitochondrial membrane
TIM23: presequence translocase of the inner mitochondrial membrane 23
TIMM: translocase of inner mitochondrial membrane protein
TMD: transmembrane domain
TOM: translocase of the outer mitochondrial membrane
TOMM: translocase of outer mitochondrial membrane protein
TPR: tetratricopeptide repeat
TRAP1: tumor necrosis factor receptor-associated protein 1
UBL: ubiquitin-like domain
UBR4: ubiquitin protein ligase E3 component N-recognin 4
ULK1: Unc-51-like autophagy-activating kinase 1
uORF: upstream open reading frame
USP30: ubiquitin-specific peptidase 30
VDAC: voltage-dependent anion channel
ΔOTC: misfolded ornithine transcarbamylase variant
Δψm: mitochondrial membrane potential
